# Supplementary figures and images for: Construction of a ceRNA network of regulated ferroptosis in doxorubicin-induced myocardial injury
Source: PeerJ. 2023 Jan 25;11:e14767. doi: 10.7717/peerj.14767 (PMC9884038; doi:10.7717/peerj.14767)

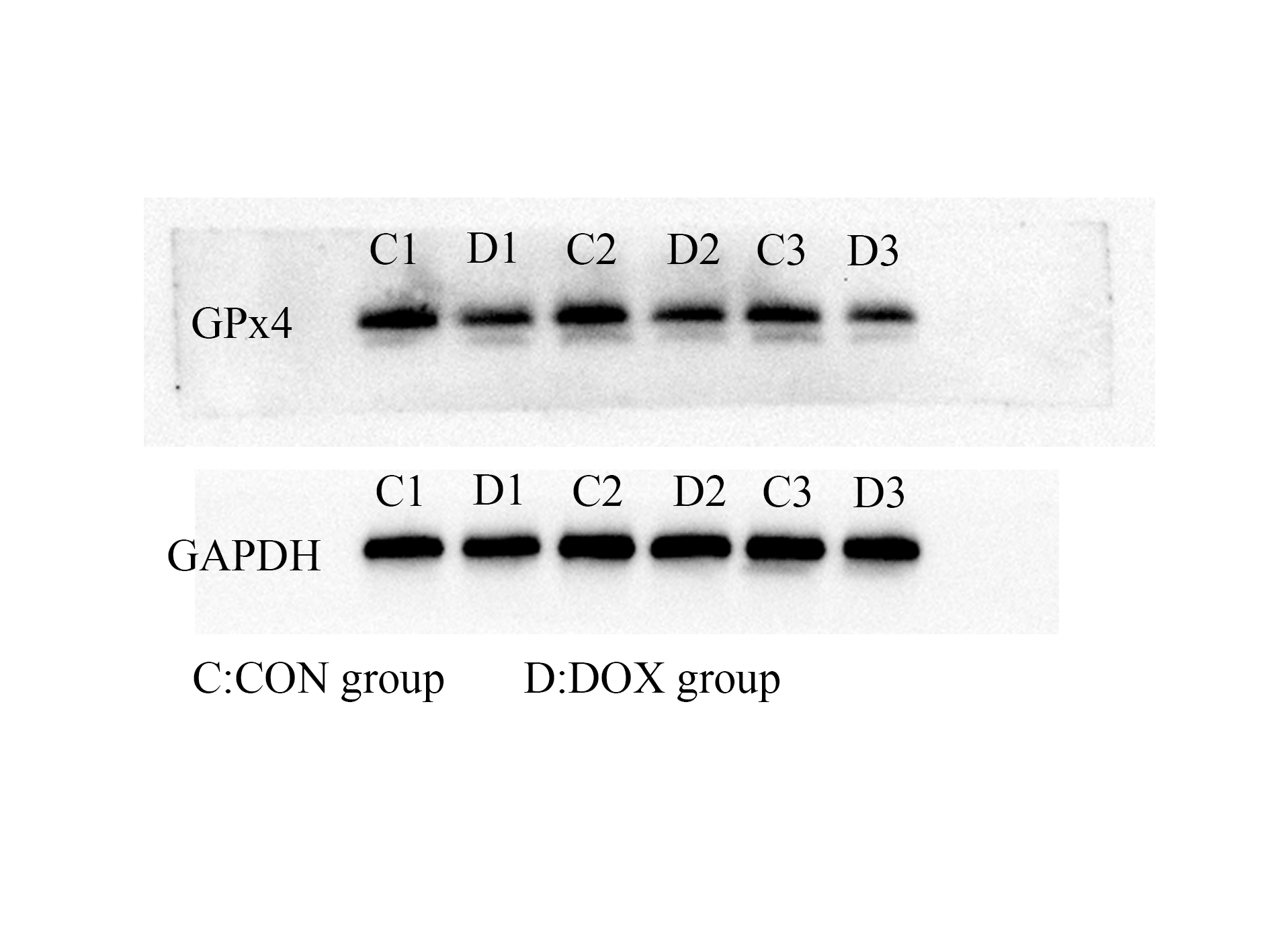

Supplement: Supplemental Information 4 [file peerj-11-14767-s004.tif]

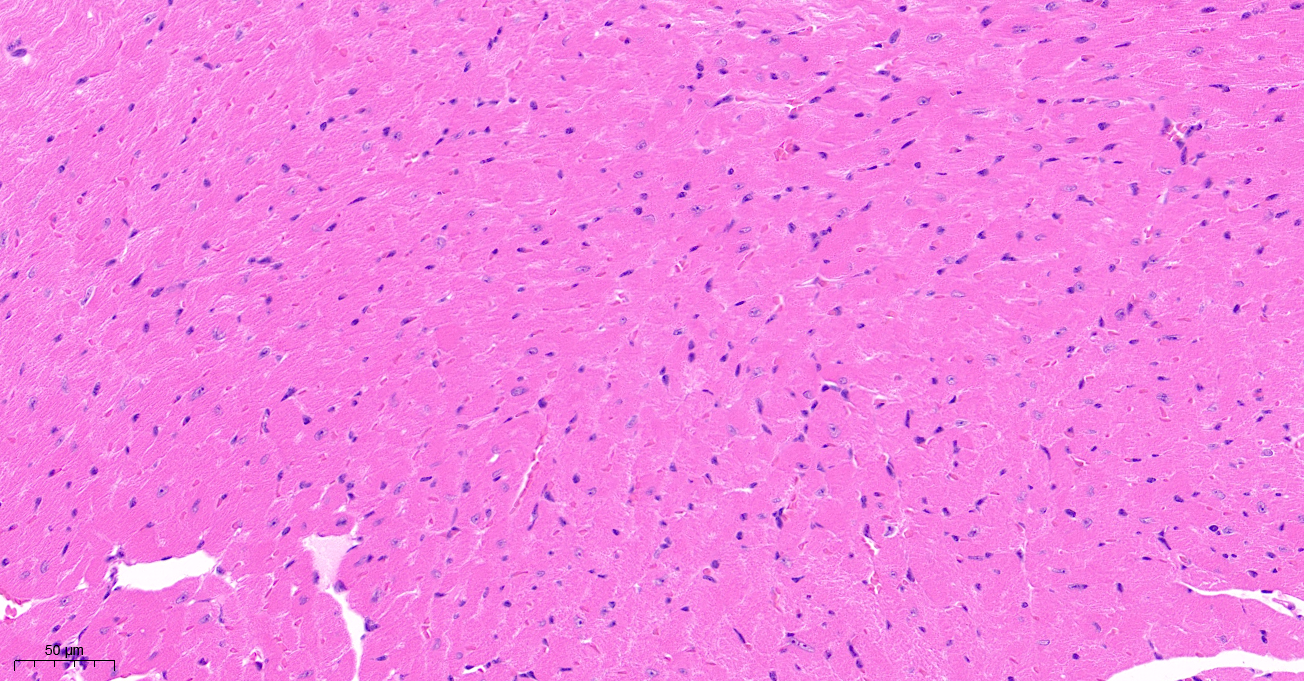

Supplement: Supplemental Information 5 [file peerj-11-14767-s005.tif]

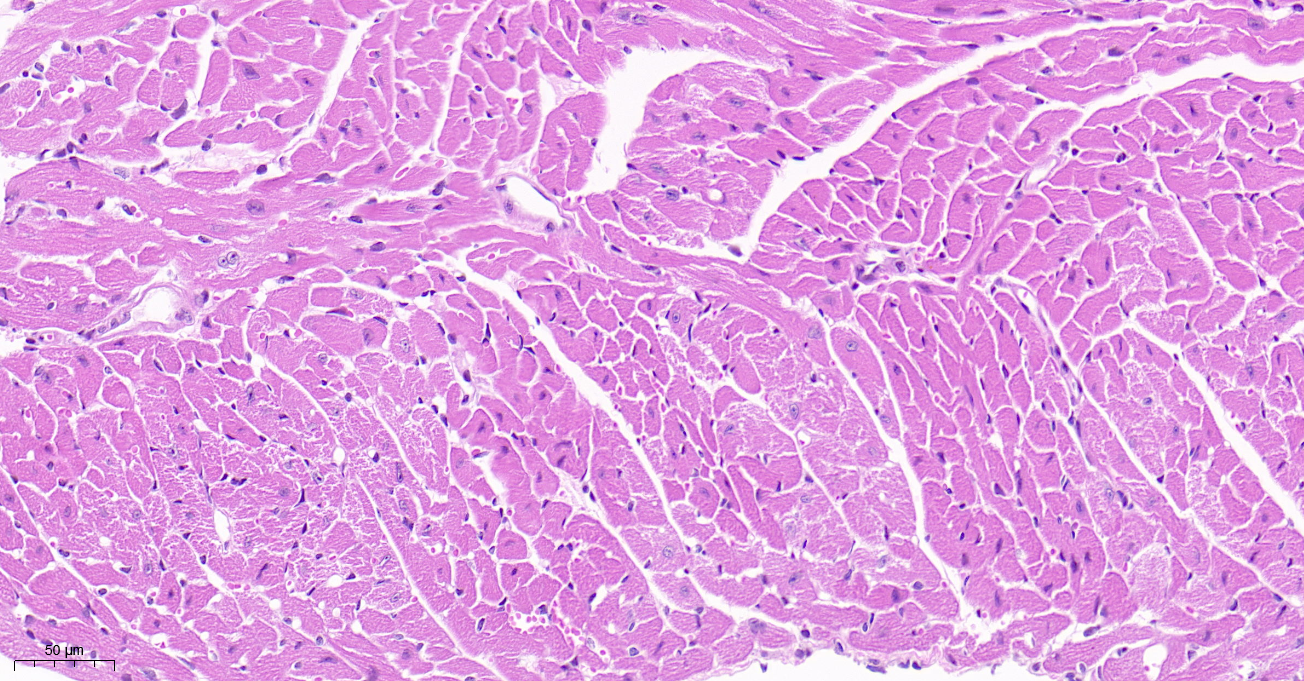

Supplement: Supplemental Information 6 [file peerj-11-14767-s006.tif]
